# Transcranial sonographic assessment of the third ventricle in neuro-ICU patients to detect hydrocephalus: a diagnostic reliability pilot study

**DOI:** 10.1186/s13613-021-00857-x

**Published:** 2021-05-04

**Authors:** Rémy Widehem, Paul Bory, Frédéric Greco, Frédérique Pavillard, Kévin Chalard, Alexandre Mas, Flora Djanikian, Julie Carr, Nicolas Molinari, Samir Jaber, Pierre-François Perrigault, Gerald Chanques

**Affiliations:** 1 Department of Anaesthesia & Critical Care Medicine, Montpellier University Hospital Center, Gui de Chauliac Hospital, Montpellier, France; 2 Department of Anaesthesia & Critical Care Medicine, Montpellier University Hospital Center, Saint Eloi Hospital, Montpellier, France; 3 Department of Statistics, Montpellier University Hospital Center, La Colombière Hospital, and Institut Montpelliérain Alexander Grothendieck (IMAG), University of Montpellier, CNRS, Montpellier, France; 4 PhyMedExp, University of Montpellier, INSERM, CNRS, Montpellier, France

**Keywords:** Neurocritical care, Third ventricle, Sonography, Hydrocephalus, Point of care

## Abstract

**Background:**

Transcranial sonography is a point-of-care tool recommended in intensive care units (ICU) to monitor brain injured patients. Objectives of the study was to assess feasibility and reliability of the third ventricle (V3) diameter measurement using transcranial sonography (TCS) compared to brain computed-tomography (CT), the gold standard measurement, and to measure the TCS learning curve. Design**:** prospective study, in a 16-bed neurological ICU in an academic hospital. Every consecutive brain injured adult patient, who required a brain CT and TCS monitoring were included. The V3 diameter was blindly measured by TCS and CT. Intraclass correlation coefficient (ICC) and Bland–Altman plot were used to assess the reliability and agreement between TCS and CT V3 measurements. Diagnosis performance of the V3 diameter using TCS to detect hydrocephalus was measured. Absolute difference between V3 measurement by residents and experts was measured consecutively to assess the learning curve.

**Results:**

Among the 100 patients included in the study, V3 diameter could be assessed in 87 patients (87%) from at least one side of the skull. Both temporal windows were available in 70 patients (70%). The ICC between V3 diameter measured by TCS and CT was 0.90 [95% CI 0.84–0.93] on the right side, and 0.92 [0.88–0.95] on the left side. In Bland–Altman analysis, mean difference, standard deviation, 95% limits of agreement were 0.36, 1.52, − 2.7 to 3.3 mm, respectively, on the right side; 0.25, 1.47, − 2.7 to 3.1 mm, respectively, on the left side. Among the 35 patients with hydrocephalus, V3 diameters could be measured by TCS in 31 patients (89%) from at least one side. Hydrocephalus was, respectively, excluded, confirmed, or inconclusive using TCS in 35 (40%), 25 (29%) and 27 (31%) of the 87 assessable patients. After 5 measurements, every resident reached a satisfactory measurement compared to the expert operator.

**Conclusion:**

TCS allows rapid, simple and reliable V3 diameter measurement compared with the gold standard in neuro-ICU patients. Aside from sparing irradiating procedures and transfers to the radiology department, it may especially increase close patient monitoring to detect clinically occult hydrocephalus earlier. Further studies are needed to measure the potential clinical benefit of this method.

*Trial registration:* ClinicalTrials.gov ID: NCT02830269.

**Supplementary Information:**

The online version contains supplementary material available at 10.1186/s13613-021-00857-x.

## Introduction

Transcranial sonography (TCS) is an important point-of-care diagnostic tool [1, 2] recommended in neurological Intensive Care Unit (ICU) patients [3–5]. It is generally used for monitoring patients suffering from subarachnoid haemorrhage [6, 7], severe traumatic brain injury [8] or severe stroke. As TCS is an accessible, non-irradiating, easy to learn and reliable tool [9], it can be repeated several times a day at the bedside in order to assess intracranial hypertension [10, 11] and to guide therapeutic decisions, by measuring the Doppler velocity of intracerebral arteries. Moreover, TCS can be used to evaluate cerebral anatomy and pathology [12]. In 1990, Bogdahn et al. described the third ventricle (V3) in 45 of 49 patients free of neurological involvement [13]. Other studies found a good sensitivity and specificity of V3 TCS measurement compared to magnetic resonance imaging or computed-tomography (CT) in non-critically ill patients with neurological diseases [14–17]. One study, published 20 years ago, measured the V3 diameter during episodes of intracranial hypertension in 28 ICU patients [18]. This preliminary study did not validate this method in comparison to the gold standard examination, i.e. computed-tomography (CT).

Obstructive hydrocephalus can occur in subarachnoid haemorrhage patient and in patient with external ventricular drain. V3 study can detect this pathological issue. A retrospective study reported a good correlation between TCS and CT in 15 patients with traumatic brain injury for the measurement of V3 diameter [19]. Feasibility and reliability of the V3 diameter measured by TCS have never been prospectively reported in a large population of neuro-ICU patients. Hence, the primary goal of this study was to determine the feasibility and reliability of TCS compared to brain CT in measuring V3 diameter in neuro-ICU patients. Secondly, the diagnosis performance to detect hydrocephalus was measured. Finally, the learning curve for measuring the V3 diameter by TCS was assessed.

## Methods

The study protocol was approved by the Ethics committee of “Comité de Protection des Personnes Sud-Méditerranée I” (ID RCB: 2016-A00749-42; Protocol Version: March 11, 2016; Consent Version: June 25, 2016), ClinicalTrials.gov ID: NCT02830269. Because the trial used non-invasive procedures along with standard care provided in French ICUs [7, 20], only a verbal consent was required from the patient or relatives, according to the French law [21]. Additional file 1: Figure S1 shows the study design.

### Patients

The study took place in the 16-bed neuro-ICU of the tertiary University Hospital of Montpellier Gui de Chauliac, France. All consecutive neuro-ICU patients ≥ 18 years old, admitted to the neuro-ICU with a brain injury, were eligible for enrolment if they underwent a brain CT planned by the bedside physicians, as well as TCS monitoring. Exclusion criteria were: a sonographic assessment of the third ventricle impossible to perform within one hour around a brain CT, or consent disclaimed.

### Patient characteristics and treatments

Patient characteristics, including weight (kg), height (m), body mass index (kg/m^2^), age, gender, type of brain injury, Glasgow Coma Scale, Simplified Acute Physiology Score 2 (SAPS2) [22], Fisher score [23] for subarachnoid haemorrhage and International Severity Score (ISS) [24] for traumatic brain injury were recorded at the time of ICU admission. At the time of the TCS exam, the following variables were recorded: systolic, diastolic and mean arterial pressures, heart rate, intraventricular or intracranial pressure (if available), body temperature, blood glucose level, oxygen plethysmography saturation, presence or absence of an external ventricular drain (EVD) or a craniotomy, mechanical ventilation parameters, arterial blood gas, natremia and haemoglobinemia. The use of sedative drugs, neuromuscular blocking agents, milrinone, nimodipine and norepinephrine was recorded. Intracranial hypertension was assessed using clinical parameters: anisocoria, mydriasis or Cushing reflex. Patient outcomes were assessed: duration of mechanical ventilation, ICU length of stay, RANKIN score and mortality at ICU discharge.

### Feasibility and reliability of TCS compared with brain CT (primary goal)

The primary goal was the feasibility and the reliability of V3 diameter measurements by TCS compared to brain CT (gold standard).

#### TCS measurements

**Fig. 1:**
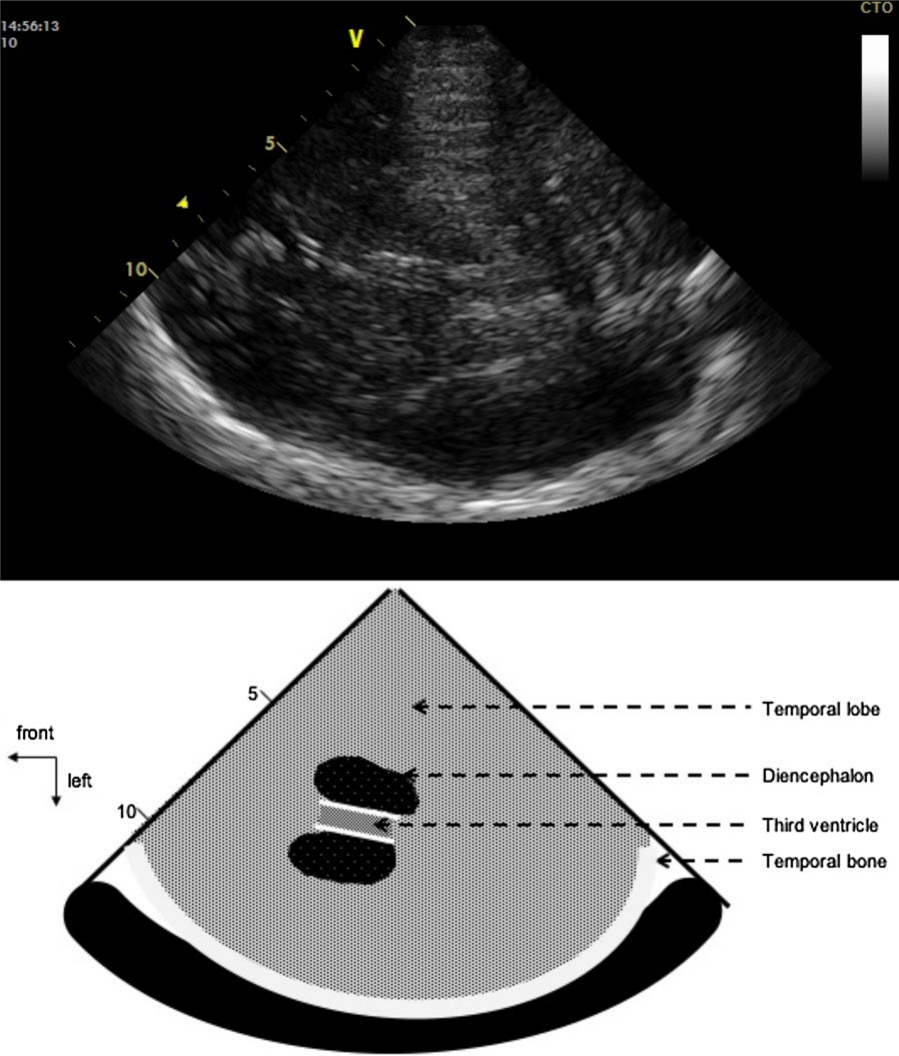
Sonographic view of V3 and the corresponding anatomic diagram. The third ventricle was identified as a double hyperechogenic image over the midbrain with the diencephalon on both sides

The measurements were performed with the patient in supine position, head 30° up, using a General Electric* Vivid-q 5–1 MHz cardiac transducer for echocardiography (GE, Boston, USA). The 2D mode was used, along with the colour and pulse waved Doppler. Both sides of the head were assessed. Cerebral blood flow velocity and pulsatility indexes were recorded on both middle cerebral arteries. The V3 diameter was measured through the temporal acoustic bone window using a low frequency (2 to 4 MHz) probe within one hour around the brain CT. The V3 was identified as a double hyperechogenic image above the midbrain with the diencephalon on both sides (Fig. 1); the V3 diameter was measured within the hyperechogenic lines. Depth was defined as the measurement between the external bone table and the first wall of V3. Measurements were performed only on optimal image planes, after zooming using the electronic calliper of the ultrasound device. An intensivist, who was trained in TCS (expert operator), measured the V3 diameter and reported the measurement on the Clinical Research Form, blinded to the brain CT measurements. Both sides of the head were assessed. Examination times were recorded.

#### Brain CT

The diameters and depth of the V3, as well as bone thickness and density, were independently and retrospectively measured by the same investigator (RW), who was blinded to the TCS measurements and to the clinical data. CT acquisition were helicoidal. Slice thickness were 1.25 mm and slice increment 0.625 mm. V3 coronal and transverse diameters were measured, as well as a “modified” diameter, which measured the diameter using the same angulation as classically used for the sonography examination view. “Modified” diameters were measured for both sides. Coronal, transverse and “modified” diameters were all measured in the larger portion of the third ventricle after zooming. Depth was measured between the external temporal bone and the outer limit of the V3. Reconstructions were made manually using coronal view, and measurements were performed on transversal view respecting both anterior/posterior commissure angulation in axial plan [25] and previously measured coronal angulation. Slice thickness and increment remained similar. All reconstructions were performed in the radiology department without files compression and data lost.

### Diagnostic performance of V3 measurement using TCS to detect hydrocephalus

Hydrocephalus was defined by a bicaudal index exceeding the upper limit of normality for the patient’s age according to a standardised method on the CT [26, 27]. V3 diameters measured by TCS were analysed secondly to define thresholds and related diagnosis performances characteristics (see “Statistics”).

## Absolute difference between V3 measurement by residents and experts

The absolute difference between the V3 measurement by residents and experts was assessed among 9 anaesthesiologist-intensivist residents at another time during the ICU stay. All residents had previous experience in TCS but not in V3 diameter measurement. Residents measured the V3 diameter, followed by an ultrasound expert ICU physician who measured the V3 during the same TCS examination. A quick explanation of the method was performed after each measurement by the expert physician. A learning curve was drawn to represent the evolution of the mean absolute difference between the V3 measurement by the residents and the V3 measurement by an expert physician for consecutive measurements. Reaching 1 mm of absolute difference between the measurements of the resident and the expert physician was determined to qualify a satisfactory skill. This difference of 1 mm was determined based on the technical characteristics of the transducer’s precision, which is 1 mm for a 5–1 MHz cardiac transducer using a low frequency (2 to 4 MHz) at 7 mm depth.

## Statistics

Data are presented as numbers (percentage) and medians with interquartile range (IQR). The 95% confidence intervals (95% CI) are given when appropriate.

### Feasibility and reliability of TCS compared with brain CT (primary goals)

The feasibility of the measurement of the V3 diameter by TCS was measured as the proportion of assessable patients.

The reliability of TCS in measuring the V3 diameter, compared to the CT measurement considered as the gold standard method [28], was evaluated by the intraclass correlation coefficient (ICC). The Bland–Altman method analysed the agreement between the two types of measurements (TCS and CT). Only “modified” V3 diameters measured on CT using the angle correction were taken into account. For the Bland–Altman, the mean difference with 95% CI, the limits of agreement at 95% CI were assessed [29]. The ICC and Bland–Altman plots were analysed separately for each side of measurement (right and left).

### Power analysis

Expecting the V3 diameter measurements using TCS and CT to have an ICC of at least 0.85 based on previous data [14–17, 19] with a half range of the 95% CI of 0.15, 59 paired measurements of TCS and CT were necessary to analyse. Expecting 10% of patients to have no V3 visible on TCS based on previous studies [15, 30], and expecting 10% to be excluded, we planned on including 100 patients.

### Diagnostic performance of V3 measurement using TCS to detect hydrocephalus

Hydrocephalus was diagnosed by CT [26, 27]. The Mann–Whitney–Wilcoxon’s test was used to compare V3 diameters between different subgroups of patients (patients with and patients without hydrocephalus). A *p* value < 0.05 was considered as significant. The risk of hydrocephalus was established from an analysis of statistical performances of the V3 TCS measurement, by the analysis of the receiver operating characteristic (ROC) curve. Using a previously published approach [31], we defined a grey zone (for which predicting hydrocephalus was not conclusive) for cut-offs with a sensitivity lower than 90% and a specificity lower than 90% (diagnosis tolerance of 10%). On either side of these cut-offs, patients were assigned to a low-risk or a high-risk.

### Absolute difference between V3 measurement by residents and experts (learning curve)

The absolute difference between V3 diameters measured by residents and expert physicians using TCS was calculated. One millimetre of absolute difference between the measurements of the resident and the expert physician defined a satisfactory skill. The mean number of assessments needed to reach this threshold was recorded. Every subsequent measurement performed by a given resident was made on a different patient.

## Results

Between August 2016 and May 2017, 100 consecutive adult patients were included. Additional file 1: Figure S2 shows the study flowchart. Table 1 and Additional file 1: Table S1 show patient characteristics and outcomes.

**Table 1:** Demographic and medical characteristics

Characteristics upon admission to ICU	*N* = 100
Age (years)	62 [52–70]
Sex (F/M)	52/48
Body mass index (kg m^−2^)	26 [22–29]
Reason for admission to the ICU	
Aneurysmal subarachnoid haemorrhage, *n* (%)	33 (33%)
Intracranial haematoma, *n* (%)	30 (30%)
Stroke, *n* (%)	13 (13%)
Head trauma, *n* (%)	5 (5%)
Post-operative patients, *n* (%)	15 (15%)
Meningoencephalitis	2 (2%)
Cardiac arrest	1 (1%)
Undetermined coma	1 (1%)
Simplified Acute Physiological Score II	40 [31–53]
Glasgow Coma Scale	7 [4–13]
Fisher score*, *n* = 33	4 [3, 4]
International Severity Score score**, *n* = 5	32 [26–38]

Characteristics upon study enrolment	*N* = 100
Time between ICU admission and enrolment, days	2 [1–5]
Therapeutics, *n* (%)	
Invasive mechanical ventilation	95 (95%)
Sedation	78 (78%)
Analgesia	78 (78%)
Vasopressors (norepinephrine)	30 (30%)
Milrinone	9 [9%]
Nimodipine	28 [28%]
Clinical hypertensive symptoms, *n* (%)	10 (10%)
Cushing reflex, *n* (%)	3 (3%)
Anisocoria, *n* (%)	3 (3%)
Mydriasis, *n* (%)	4 (4%)
External ventricular derivation, *n* (%)	37 (37%)
Craniotomy, *n* (%)	14 (14%)
Duration of mechanical ventilation, days	17 [7–28]
ICU length of stay, days, *n* (%)	21 [9–37]
RANKIN score at ICU discharge	4 [3–6]
Mortality in ICU, *n* (%)	25 (25%)

Continuous data are expressed in median [25th–75th percentiles]

*ICU* intensive care unit

*Fisher score was calculated for the 33 patients with aneurysmal subarachnoid haemorrhage

**International Severity Score was calculated for the 5 patients with brain trauma

CT and TCS findings are shown in Table 2 and Additional file 1: Table S2. Hydrocephalus was reported in 35 patients. The middle cerebral artery was identified on TCS in 78 patients. Systolic, diastolic and mean cerebral artery velocities were 92 cm/s (IQR 76–120), 28 cm/s (IQR 22–40) and 48 cm/s (IQR 37–64), respectively. The median pulsatility index (PI) was 1.2 (IQR 1.1–1.6). TCS examination including middle artery Doppler and V3 assessment was performed in 7 (IQR 5–10) min. Detailed data regarding Doppler measurements are shown Additional file 1: Table S2.

**Table 2:** CT and TCS findings

	CT	TCS
Bone thickness, mm	3 [2.2–4]	NA
Radiodensity, Hounsfield unit	1204 [975–1377]	NA
Time between TCS and CT, min	NA	38 (25–52)
V3 assessable on both sides, *n* (%)	100 (%)	70 (70%)
V3 assessable on one side, *n* (%)	100 (%)	87 (87%)
Right side§		
V3 assessable, *n* (%)	100 (100%)	79 (79%)
V3 diameter, mm	6.4 [4.4–9.2]	5.8 [4.6–8.4]
Angle correction	14 [11–18]	NA
V3 depth (bone to V3), mm	65 [62–69]	72 [68–76]
Left side§		
V3 assessable, *n* (%)	100 (100%)	78 (78%)
V3 diameter, mm	6.3 [4.6–9.6]	5.8 [4.4–8.6]
Angle correction	13 [10–16]	NA
V3 depth, mm	65 [62–68]	71 [68–75]
Other CT findings		
V3 coronal diameter, mm	6.0 [4.4–9.0]	NA
V3 haematoma, *n* (%)	23 (23%)	NA
Hydrocephalus, *n* (%)	35 (35%)	NA
Hydrocephalus management, *n*/*N* (%)	8/35 (23%)	NA
External ventricle drain reopening, *n*	3	NA
External ventricle drain level adaptation, *n*	4	NA
External ventricle drain placement, *n*	1	NA
Medication, *n*	1	NA

Continuous data are expressed in median [25th–75th percentiles]

§V3 CT diameters were “modified” diameters to take into account the usual angulations of the ultrasound probe

The sum differs from 8 because one patient received several therapeutics

*CT* computed-tomography; *NA* not applicable; *TCS* transcranial sonography; *V3* third cerebral ventricle

### Feasibility and reliability of TCS compared with brain CT (primary goal)

The V3 diameter could be measured by TCS on at least one side in 87 patients (87%) and on both sides in 70 patients (70%). The V3 diameter could not be measured in 13 patients because of the impossibility to recognise the V3, in any side. “Modified” V3 diameters using the angle correction, measured by CT (gold standard), were 6.4 (IQR [4.4–9.2]) mm from the right side, and 6.3 (IQR [4.6–9.6]) mm from the left side. V3 diameters measured by TCS were 5.8 (IQR [4.6–8.4]) mm for the right side, and 5.8 (IQR 4.4–8.6]) mm for the left side.

**Fig. 2:**
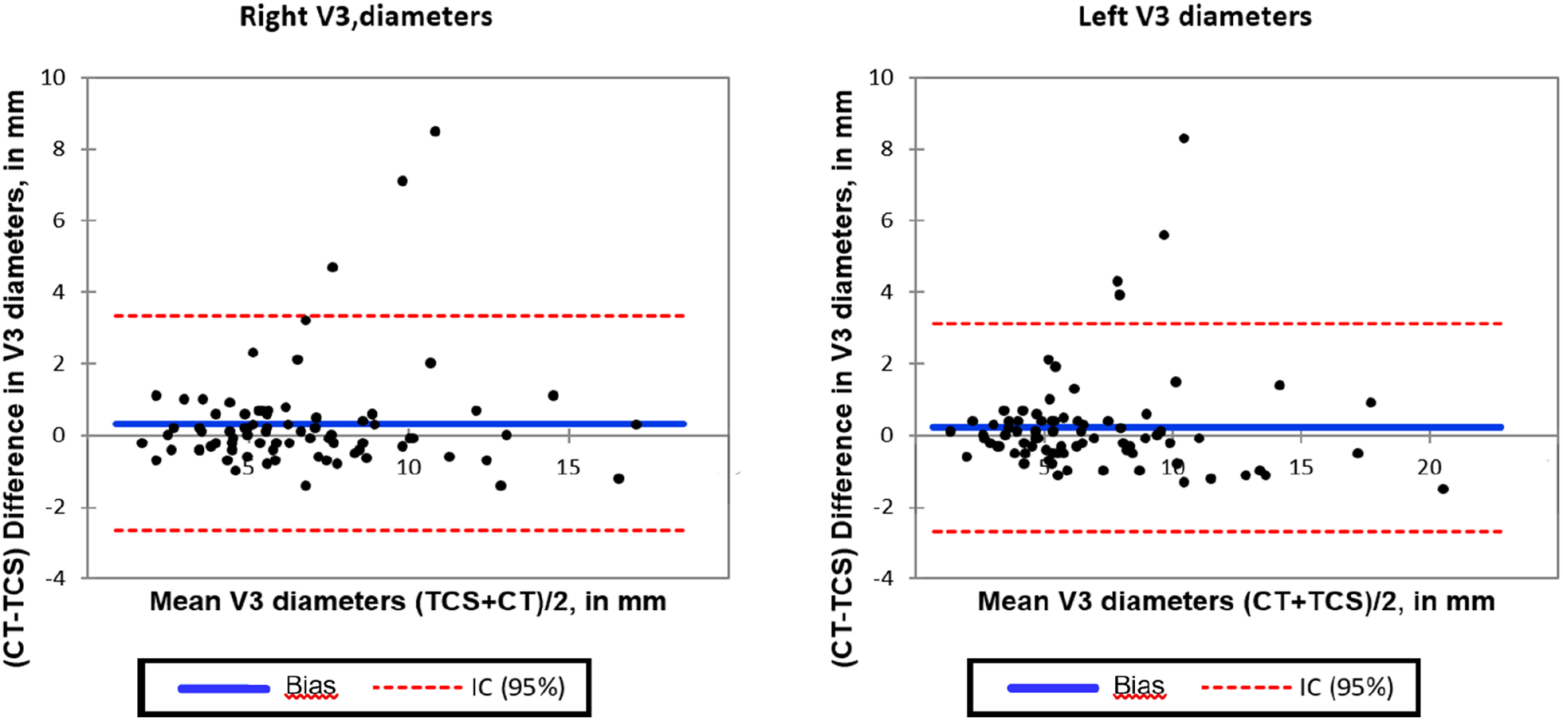
V3 diameters measured by TCS and CT according to Bland–Altman diagram. Bland–Altman plots show the agreement between TCS and CT for V3 diameter measurements for each side of measurement. Bars lines represent 95% limits of agreement, and blue lines represent the mean difference. Mean difference between CT and TCS was 0.36 mm (standard deviation (SD) 1.52 mm, 95% limits of agreement (95% LOA) − 2.7 mm to 3.3 mm) for the right side, and 0.25 mm (SD 1.47 mm, 95% LOA − 2.7 mm to 3.1 mm) for the left side

The ICC between TCS and CT were 0.90 (95% CI 0.84 to 0.93) for the right side and 0.92 (CI 0.88 to 0.95) for the left side (Additional file 1: Figure S3). Figure 2 shows the Bland–Altman analysis diagram. Mean difference between CT and TCS was 0.36 mm (standard deviation (SD) 1.52 mm, 95% limits of agreement (95% LOA) − 2.7 mm to 3.3 mm) for the right side, and 0.25 mm (SD 1.47 mm, 95% LOA − 2.7 mm to 3.1 mm) for the left side. For the right side, the 95% CI of the mean difference was 0.36 (0.02 to 0.7) mm and the 95% CI of the lower and upper limits of agreement were − 2.7 (− 3.28 to − 2.12) mm and 3.3 (2.72 to 3.88) mm, respectively. For the left side, 95% of the mean difference was 0.25 (− 0.08 to 0.58) mm and the 95% CI of the lower and upper limits of agreement were − 2.7 (− 3.27 to − 2.13) mm and 3.1 (2.53 to 3.67) mm, respectively.

## Diagnostic performance of V3 measurement using TCS to detect hydrocephalus

**Fig. 3:**
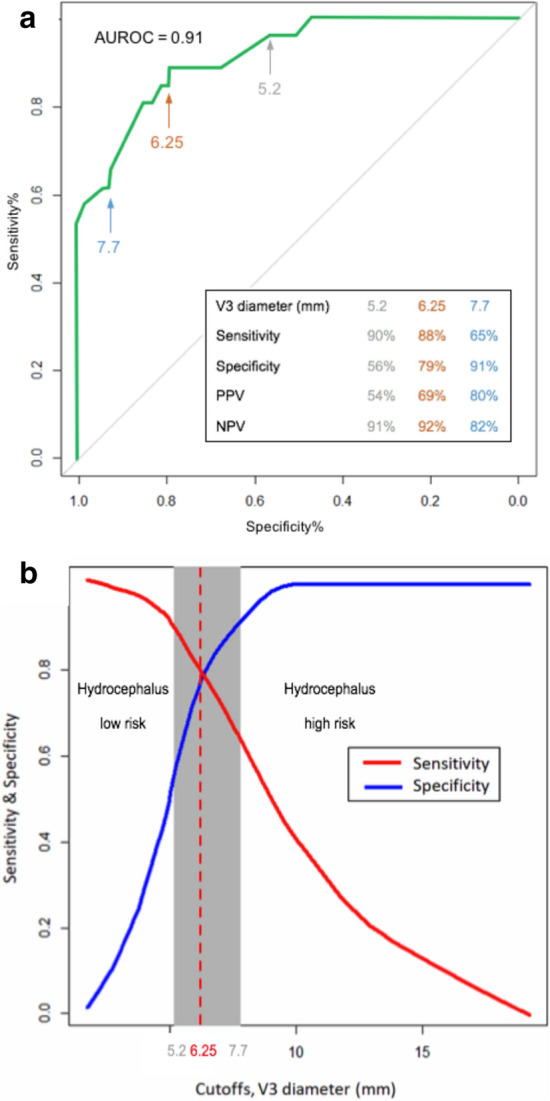
**a** Receiver operating characteristic (ROC) curve of V3 diameter measured by TCS to detect hydrocephalus diagnosed on CT. The ROC curve represents the specificity and sensitivity of TCS to diagnose hydrocephalus. AUC was 0.91% [95% CI 0.85–0.98%], the estimated best threshold was at 6.25 mm, with a specificity of 0.79 and a sensibility of 0.88. Positive predictive value (PPV) and negative predictive value (NPV) are provided for thresholds on either side of the grey zone (see Fig. 3b). **b** Two-curve sensitivity/specificity representation of grey zone for V3 diameter measured by TCS, associated with a hydrocephalus diagnosed on CT. We defined a grey zone for cut-offs with a sensitivity lower than 90% and a specificity lower than 90% (diagnosis tolerance of 10%). On either side of these cut-offs, patients were assigned to a low-risk or a high-risk. The diagnosis of hydrocephalus was thus excluded in 35 (40%) patients with a V3 diameter < 5.2 mm and confirmed in 25 (29%) patients with a V3 diameter > 7.7 mm. TCS was inconclusive in 27/87 (31%) patients. The V3 measurement from the right side was included for these analyses because right side was the most frequently assessable side. The left side was included when V3 was not assessable from right side

The V3 was assessable by TCS in 31 (89%) patients among the 35 patients with hydrocephalus diagnosed using the CT. The median V3 diameter measured by TCS was significantly greater in the hydrocephalus group (8.8 (IQR 6.8 to 11.7) mm) compared to the non-hydrocephalus group (5.0 (IQR 3.8 to 6.1) mm, *p* < 0.0001) (Additional file 1: Figure S4). The ROC curve analysis for V3 diameter measured by TCS had an AUC of 0.91 [95% CI 0.85–0.98]. The best threshold of V3 diameter associated with hydrocephalus diagnosis was 6.25 mm, corresponding to a specificity of 0.79 and a sensibility of 0.88 (Fig. 3a). Figure 3b shows the two-graph ROC curves for sensitivity and specificity defining an inconclusive zone (grey zone). TCS was inconclusive (sensitivity or specificity less than 90%) in 27/87 patients (31%) for a V3 diameter between 5.2 and 7.7 mm. The diagnosis of hydrocephalus was thus excluded in 35 (40%) patients with a V3 diameter < 5.2 mm and confirmed in 25 (29%) patients with a V3 diameter > 7.7 mm.

The V3 measurement from the right side was included for these analyses because right side was the most frequently assessable side. The left side was included when V3 was not assessable from right side.

## Absolute difference between V3 measurement by residents and experts (secondary end point)

**Fig. 4:**
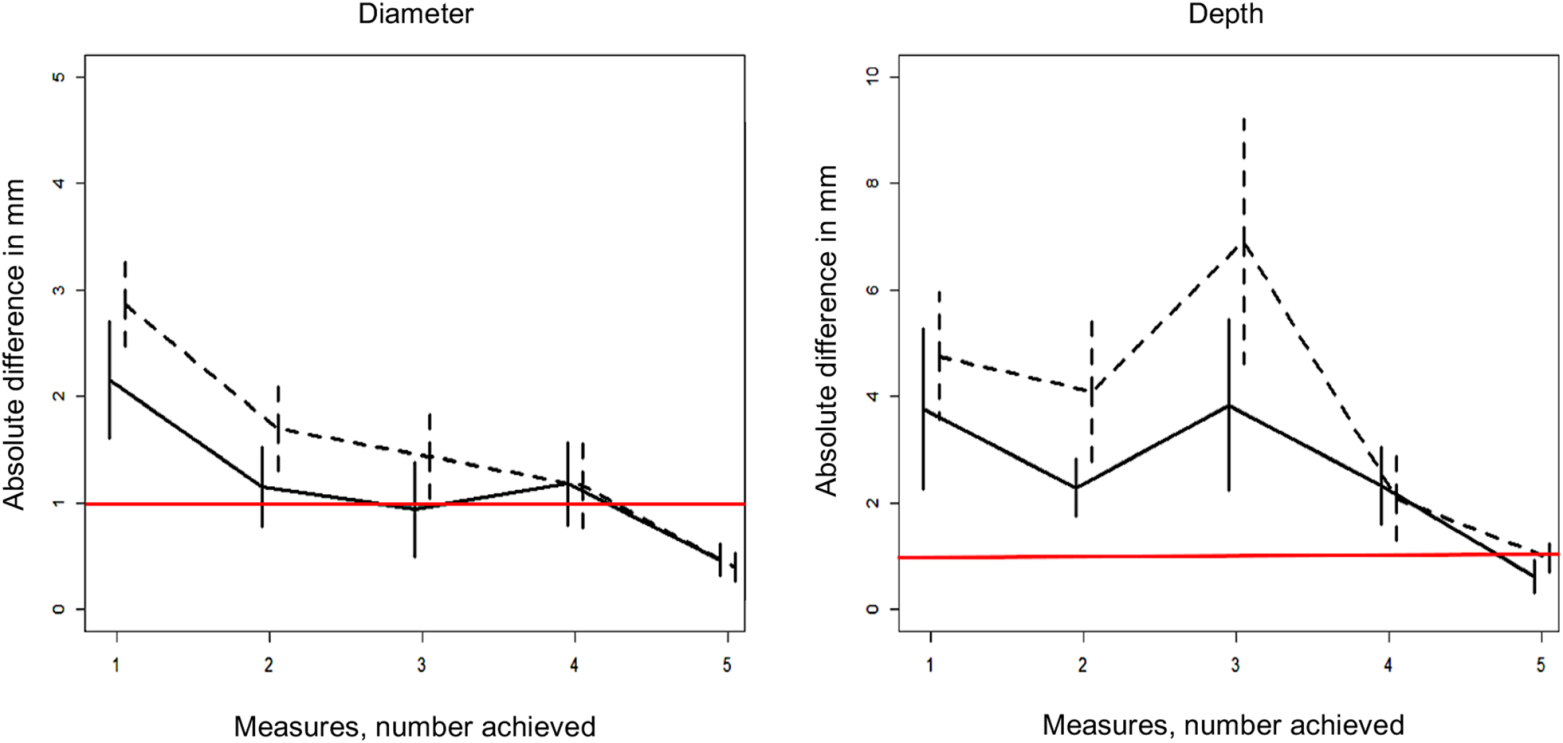
Learning curves for V3 measurement by TCS. Plots represent the mean absolute difference between the V3 measurement by the 9 participating residents and the V3 measurement by an expert physician. Bars represent the standard errors of the mean. Full line represents right side, and dotted line the left side. Red line represents the 1-mm threshold which was the a priori defined learning objective

Figure 4 shows the absolute difference between the V3 measurement by residents and experts, representing a learning curve for the ability to measure the V3 diameter using TCS. Forty-five measurements were performed on 31 patients by 9 residents. After 5 measurements, all residents reached a satisfactory measurement compared to the expert physician.

## Discussion

### Main findings

Feasibility was good, with 87 (87%) of patients amenable to TCS measurements, and the reliability was high compared to the gold standard (brain CT). TCS was effective to exclude or confirm hydrocephalus in two-thirds of patients at bedside. Moreover, the learning curve is steep, with only 5 procedures necessary to acquire a satisfactory skill.

### Relation with existent literature

Our findings are consistent with the existing literature concerning non-ICU patients. Regarding the feasibility of TCS to identify the V3, Bogdahn et al. [13] described V3 measurement in 45 of 52 subjects without neurological involvement (87%). More recently, Schminke et al. [15] described V3 measurement in 27 of 29 subjects with multiple sclerosis (93%). The V3 diameter was 4.4 [± 1.7] mm in the study by Schminke et al. [15], while Puz et al. [32] found similar values with a median V3 diameter of 4.5 (IQR 3.8–5.4) mm in 20 healthy volunteers. In the present study, neuro-ICU patients had a median V3 diameter of 6.5 mm [4.4 to 9.1]. The larger V3 diameters in the present study may be explained by the differences in patients pathologies and ages, V3 enlarging with age [33]. The mean age of patients included in the present study was higher than for the patients included in the two previous studies [15, 32] (59 versus 37 years). Bendella et al*.* described similar V3 diameters than us in patients with a mean age of 56 years after a decompressive craniectomy [34].

V3 diameter measurement by TCS compared to CT, is consistent with the findings of the recent retrospective study by Oliveira et al. [19] that showed an ICC of 0.85. However, in that study, the V3 diameters were greater than 30 mm and limits of agreement were very large (IQR − 14.97 to 14.81 mm). This could be explained by the retrospective nature of this study, the absence of a standardised measurement of the V3 by CT, as well as the small number of patients (*n* = 15).

### Clinical implications

TCS has a pivotal role in the ICU: it is a non-invasive, reproducible and bedside available tool, with a low cost. In patients with an adequate acoustic window (almost 9 patients out of 10), it allows for a non-irradiating assessment of the brain parenchyma and cerebral blood flow [35] without the risks of repeated CT scans, especially regarding the risk of transport and monitoring of patients to the CT room, as well as the risk of EVD disconnection related to the patient mobilisation [36].

The V3 diameter was measurable in 87 (87%) of our patients and, compared to the CT, was conclusive in 60/87 patients (69%) for a V3 diameter < 5.2 or > 7.7 mm for diagnosing hydrocephalus (Fig. 4). Therefore, TCS provides a reliable information on the diagnosis of hydrocephalus for 60 patients (60%) and offers the clinician a follow-up of the V3 diameter, without the risk of repeated CT scans.

TCS could be helpful to monitor EVD management and weaning at the bedside, or to detect clinically occult hydrocephalus. EVD management impacts the ICU and hospital lengths of stay in patients with aneurysmal subarachnoid haemorrhage [37]. In 2016, the American Neurocritical Care Society released recommendations to encourage EVD weaning “as quickly as clinically feasible” [38]. Despite this recommendation, Chung et al. showed that EVD weaning was mainly gradual over 4 days in the USA [39]. During EVD weaning, patients may have rapid change of the V3 diameter. This is usually suspected when a clinical worsening is observed and confirmed only by brain CT. The clinical examination during EVD weaning can be difficult, especially in unconscious ICU patients. Indeed, the neurological examination of sedated patients is challenging. Despite the fact that ICU patients are nowadays less sedated [40–43], one of the last indications for a deep or prolonged sedation is for brain injured patients [44]. According to the present study, TCS might allow an easy and safe screening of clinically occult hydrocephalus that can be performed systematically and repeatedly at the bedside. The present study showed that TCS can precisely detect patients with or without hydrocephalus in 69% of the cases when the V3 is assessable (Fig. 3). Further studies based on V3 measurement and TCS Doppler are necessary before implementing such a strategy determining the proportion of patients for whom TCS could shorten the time of recognition of hydrocephalus as well as the time to intervention. In many patients, a confirmation using CT is mandatory. TCS could also detect an early increase of the V3 diameter during EVD weaning. TCS provides an enhanced monitoring of brain injured patients and allows performing CT or therapeutic change quickly. In neurocritical care, time is brain, and a diminution of the duration of brain injury related to acute hydrocephalus could improve patient outcome. Such strategy remains to be evaluated by further studies. However, implementing V3 measurements by TCS requires the availability of this technique as well as a sufficient training. Other indications of TCS to localise cerebral ventricles in the future could be to help inserting DVE, but this procedure needs further investigation.

Every anaesthesiologist-intensivist resident with previous TCS Doppler experience but without V3 sonography experience could reach satisfactory skill after only five measurements. Four residents out of 9 made a substantial error on the third measurement. Therefore, even if the second measure is close to that of the expert operator, five measurements seem necessary to reach satisfactory skill. Measuring the V3 diameter by TCS appears to be an easy skill to learn. This tool could become widespread over ICUs after a short training by expert TCS operators. If the relevance of V3 monitoring is confirmed, it could be implemented as a first line strategy and interest centres where brain CT is not quickly available.

Strengths of this study are that it is the first prospective study, in a large population of neuro-ICU patients with diverse brain illnesses, which compared TCS and CT on V3 measurements, each investigator blinded to one other, with standardised measurements for both TCS and CT. Furthermore, the study had a high inclusion rate of consecutively admitted patients within a short period of time (8 months). This suggests the feasibility of TCS in routine.

Our study has several limitations. First, we were unable to measure V3 diameter with TCS in 13 patients (13%) because of the impossibility to recognise the V3 using echography. The risk of failure using echography in critically ill patients is one of the main issues related to this technique, as also describe for other indications such as cardiac (1% of failure [45]) or pneumothorax (12% of failure [46]) assessments. Secondly, we did not directly assess intra- and inter-observer reliabilities. Regarding the intra-observer reliability, it can be estimated comparing measurements on both sides in the 70 patients with bilateral V3 visualisation. The ICC was 98% suggesting a good intra-observer reliability (Additional file 1: Figure S5). These findings are consistent with the literature, Schminke et al. showing an intra-observer ICC of 99.4% [15]. Regarding the inter-observer reliability, the study was not designed to test this property, but to assess the training of residents. The learning curves could reflect indirectly inter-observer reliability that was good after only a short training period. In all, V3 diameter measurement by TCS seems to be a reliable tool. Finally, we could not compare the time taken by the residents to the time taken by the experts to perform the measures. This is because the time that was measured for the experts included the V3 measurement as well as the Doppler velocities (accuracy study). To measure the V3 diameter, the residents took a median of 7 (IQR [5 to 8]) min and 5 (IQR [3 to 7]) min at their first and last attempt, respectively, suggesting a skill improvement (learning curve).

In conclusion, TCS is a feasible, simple, reliable and non-invasive tool for measuring V3 diameters in neuro-ICU patients at the bedside and is associated with a short learning curve. Monitoring V3 diameters by TCS could be a point-of-care tool to facilitate the early diagnosis of hydrocephalus. The role of TCS for the measurement of the V3 diameter in the management and weaning of EVD requires further studies.

## Supplementary Information

**Additional file1**: **Table S1**: Patient characteristics, **Table S2**: CT and TCS findings for the 100 patients included for analysis, **Figure S1**: Study design, **Figure S2**: Study flow chart, **Figure S3**: Right and left sides V3 diameters measured by TCS compared with CT, **Figure S4**: Comparison of V3 diameters measured by TCS, according to the hydrocephalus status, **Figure S5**: V3 diameters measured by TCS compared with the other side

## Data Availability

The datasets used and analysed during the current study are available from the corresponding author on reasonable request. Preliminary data of this study were presented at the annual meeting of the French Society of Anaesthesia and Intensive Care Medicine, 21–23 September 2017, Paris.

## Abbreviations

ICU: Intensive care unit
TCS: Transcranial sonography
CT: Computed-tomography
V3: Third ventricle
ICC: Intraclass correlation coefficient
APS2: Simplified Acute Physiology Score 2
ISS: International Severity Score
EVD: External ventricular drain
IQR: Interquartile range
95% CI: 95% Confidence interval
ROC: Receiver operating characteristic
PI: Pulsatility index
