# Supplementary material for: Transcranial sonographic assessment of the third ventricle in neuro-ICU patients to detect hydrocephalus: a diagnostic reliability pilot study
Source: Ann Intensive Care. 2021 May 4;11:69. doi: 10.1186/s13613-021-00857-x (PMC8096880; doi:10.1186/s13613-021-00857-x)
Supplement: Supplementary file 1 — Additional file1: Table S1: Patient characteristics, Table S2: CT and TCS findings for the 100 patients included for analysis, Figure S1: Study design, Figure S2: Study flow chart, Figure S3: Right and left sides V3 diameters measured by TCS compared with CT, Figure S4: Comparison of V3 diameters measured by TCS, according to the hydrocephalus status, Figure S5: V3 diameters measured by TCS compared with the other side [file 13613_2021_857_MOESM1_ESM.pdf]

Supplementary Appendix

This appendix has been provided by the authors to give readers additional informations about their work.
Supplement to: Widehem et al. Transcranial sonographic assessment of the third ventricle in neuro-ICU patients to detect hydrocephalus: a diagnostic reliability pilot study.
ClinicalTrials.gov number, NCT02830269

Table A1: Patient characteristics (page 2)

Table A2: CT and TCS findings for the 100 patients included for analysis (page 4)

Figure A1: Study design (page 5)

Figure A2: Study flow chart (page 6)

Figure A3: Figure A3: Right and left sides V3 diameters measured by TCS compared with CT (page 7)

Figure A4: Comparison of V3 diameters measured by TCS, according to the hydrocephalus status (page 8)

Figure A5: V3 diameters measured by TCS compared with the other side (page 9)

Table A1: Patient characteristics

**Characteristics upon admission to ICU**	n=100
Age (years)	62 [52-70]
Sex (F/M)	52/48
Body Mass Index ($kg.m^{-2}$)	26 [22-29]
Reason for admission to the ICU	
Aneurysmal subarachnoid haemorrhage, n (%)	33 (33%)
Intracranial Hematoma, n (%)	30 (30%)
Stroke, n (%)	13 (13%)
Head trauma, n (%)	5 (5%)
Post-operative patients, n (%)	15 (15%)
Meningoencephalitis	2 (2%)
Cardiac arrest	1 (1%)
Undetermined Coma	1 (1%)
Simplified Acute Physiological Score II	40 [31-53]
Glasgow Coma Scale	7 [4-13]
Fisher score*, n=33	4 [3-4]
International severity score score**, n=5	32 [26-38]
**Characteristics upon study enrolment**	
Time between ICU admission and enrolment, days	2 [1-5]
**Physiological parameters**	
Heart Rate (b/min)	72 [62-82]
Systolic arterial pressure (mmHg	140 [121-159]
Diastolic arterial pressure (mmHg)	68 [60-73]
Mean arterial pressure (mmHg)	91 [81-105]
Respiratory Rate (b/min)	16 [14-19]
Oxygen saturation (%)	100 [98-100]
Temperature (°C)	37.0 [36.4-37.5]
Intra ventricular Pression, (mmHg)	10 [7-11]
Cerebral perfusion Pression, (mmHg)	79 [68-87]
Glycemic blood level (g/L)	1.2 [1.1-1.5]
**Biological parameters**	
pH	7.43 [7.40-7.50]
PaCO2, (mmHg)	36 [32-38]
PaO2, (mmHg)	111 [96-128]
HCO3-, (mmol/L)	24 [22-26]
Sodium, (mmol/L)	140 [137-142]
Hemoglobinemia, (g/dL)	12 [10-13]
**Invasive mechanical ventilation, n (%)**	95 (95%)
Assist Control Volume, n/N (%)	79/95 (83%)
Pressure Support Ventilation, n/N (%)	16/95 (17%)
Tidal volume (ml/kg of Ideal Body Weight)	7.2 [6.7-7.7]
Positive End Expiratory Pressure (cm H2O)	5 [5-6]
**Pharmacological parameters**	
Sedation, n (%)¶	78 (78%)
Midazolam, n/N (%)	74/78 (95%)
Dose ($µg.kg^{-1}.min^{-1}$)	3.3 [2.3-4.2]
Propofol, n/N (%)	5/78 (6%)
Dose ($µg.kg^{-1}.min^{-1}$)	25 [20-60]
Analgesia, n (%)	78 (78%)
Sufentanil, n/N (%)	78/78 (100%)
Dose ($µg.kg^{-1}.h^{-1}$)	0.2 [0.14-0.25]

Table A1 – To be continued

Vasopressors (norepinephrine), n (%)	30 (30%)
Dose (µg.kg^{-1}.min^{-1})	0.2 [0.1-0.4]
Milrinone, n (%)	9 [9%]
Dose, (mg/h)	4.4 [4-5.7]
Nimodipine, intravenous administration, n (%)	18 [18%]
Dose, (mg/h)	1.6 [1-2]
Nimodipine, oral administration, n (%)	10 [10%]
Dose, (mg/day)	300 [360-360]
**Clinical hypertensive symptoms, n (%)**	10 (10%)
Cushing reflex, n (%)	3(3%)
Anisocoria, n (%)	3(3%)
Mydriasis, n (%)	4(4%)
External ventricular derivation, n (%)	37 (37%)
Craniotomy, n (%)	14 (14%)
Duration of mechanical ventilation, days	17 [7-28]
ICU Length of stay, days	21 [9-37]
RANKIN score at ICU discharge	4 [3-6]
**Mortality in ICU, n (%)**	25 (25%)

Continuous data are expressed in median [25th-75th percentiles].

*Fisher score was calculated for the 33 patients with aneurysmal subarachnoid haemorrhage.

**International severity score was calculated for the 5 patients with brain trauma.

ICU: Intensive Care Unit;

¶ Percent sum differs from 100% because one patient received several sedatives

Table A2: CT and TCS findings for the 100 patients included for analysis

	CT	TCS
Bone thickness, mm	3 [2.2-4]	NA
Radiodensity, Hounsfield unity	1204 [975-1377]	NA
Time between TCS and CT, min	NA	38 (25-52)
Examen duration*, min	NA	7 (5-10)
V3 assessable on both sides, n (%)	100 (%)	70 (70%)
V3 assessable on one side, n (%)	100 (%)	87(87%)
**Right side**		
V3 assessable, n (%)	100 (100%)	79 (79%)
V3 diameter, mm	6.4 [4.4-9.2]	5.8 [4.6-8.4]
Angle correction, °	14 [11-18]	NA
V3 depth (bone to V3), mm	65 [62-69]	72 [68-76]
MCA assessable, n (%)	NA	70 (70%)
MCA, systolic pressure cm/s	NA	92 (70-121)
MCA, diastolic pressure cm/s	NA	30 (22-41)
MCA, mean pressure cm/s	NA	48 (35-68)
Pulse Index	NA	1.2 (1.1-1.6)
**Left side**		
V3 assessable, n (%)	100 (100%)	78 (78%)
V3 diameter, mm	6.3 [4.6-9.6]	5.8 [4.4-8.6]
Angle correction, °	13 [10-16]	NA
V3 depth, mm	65 [62-68]	71 [68-75]
MCA assessable, n (%)	NA	69 (69%)
MCA, systolic pressure cm/s	NA	89 (77-114)
MCA, diastolic pressure cm/s	NA	28 (21-39)
MCA, mean pressure cm/s	NA	48 (38- 60)
Pulse Index	NA	1.2 (1-1.5)
Other CT findings		
V3 Coronal diameter, mm	6.0 [4.4-9.0]	NA
V3 hematoma, n (%)	23 (23%)	NA
**Hydrocephalus, n (%)**	35 (35%)	NA
**Hydrocephalus management, n/N (%)**	8/35 (23%)	NA
External ventricle drain reopening, n¶	3	NA
External ventricle drain level adaptation, n¶	4	NA
External ventricle drain placement, n¶	1	NA
Medication, n¶	1	NA

Continuous data are expressed in median [25th-75th percentiles].

CT: Computed Tomography; TCS: Transcranial sonography; MCA: Mild cerebral arteria; NA: Not Applicable.

* TCS procedure duration included all the procedure: middle cerebral artery Doppler and V3 diameter measurement

¶The sum differs from 8 because one patient received several therapeutics.

Figure A1: Study design

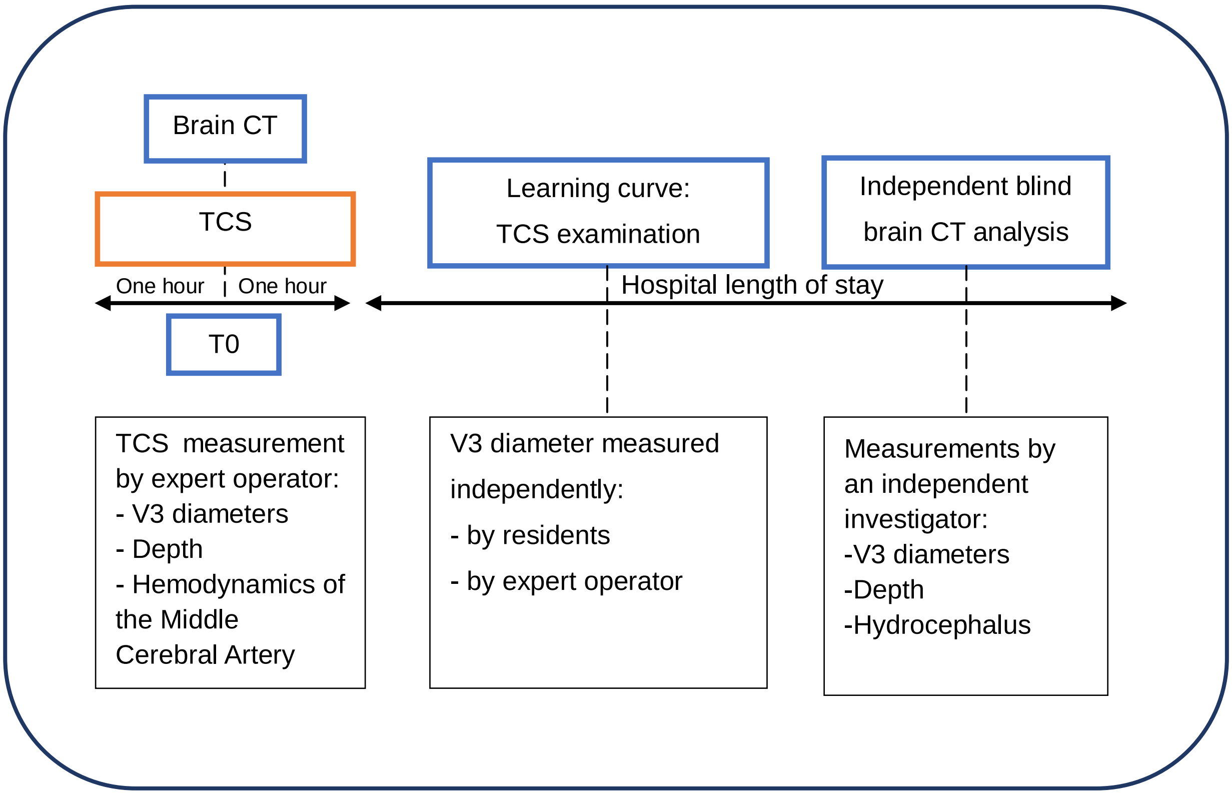

CT: computed tomography; TCS: transcranial sonography; V3: third cerebral ventricle

Figure A2: Study flow chart

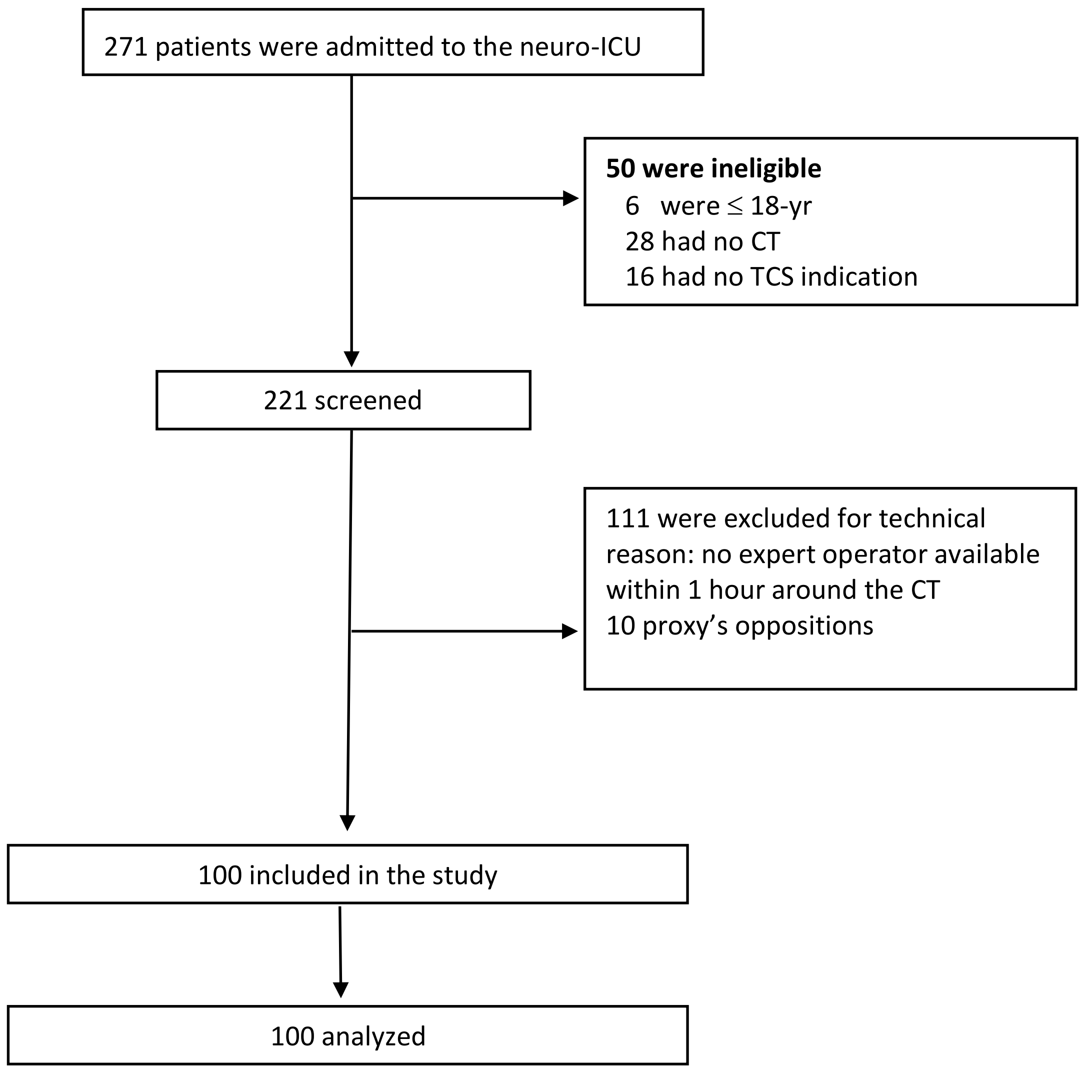

CT: computed tomography; TCS: transcranial sonography

Figure A3: Right and left sides V3 diameters measured by TCS compared with CT.

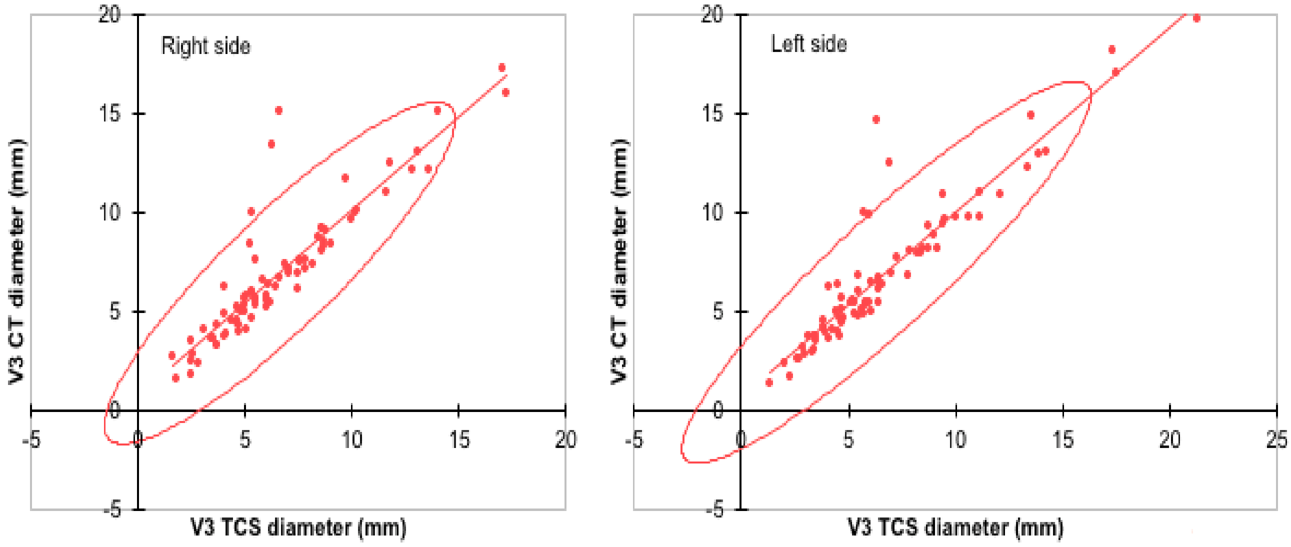

V3 diameters measured with TCS, compared with CT. The intraclass correlation between TCS and CT were 0.90 (95%CI 0.84 to 0.93) for the right side and 0.92 (CI 0.88 to 0.95) for the left side. The ellipse represents the confident interval (CI) 95%.

Figure A4: Comparison of V3 diameters measured by TCS, according to the hydrocephalus status.

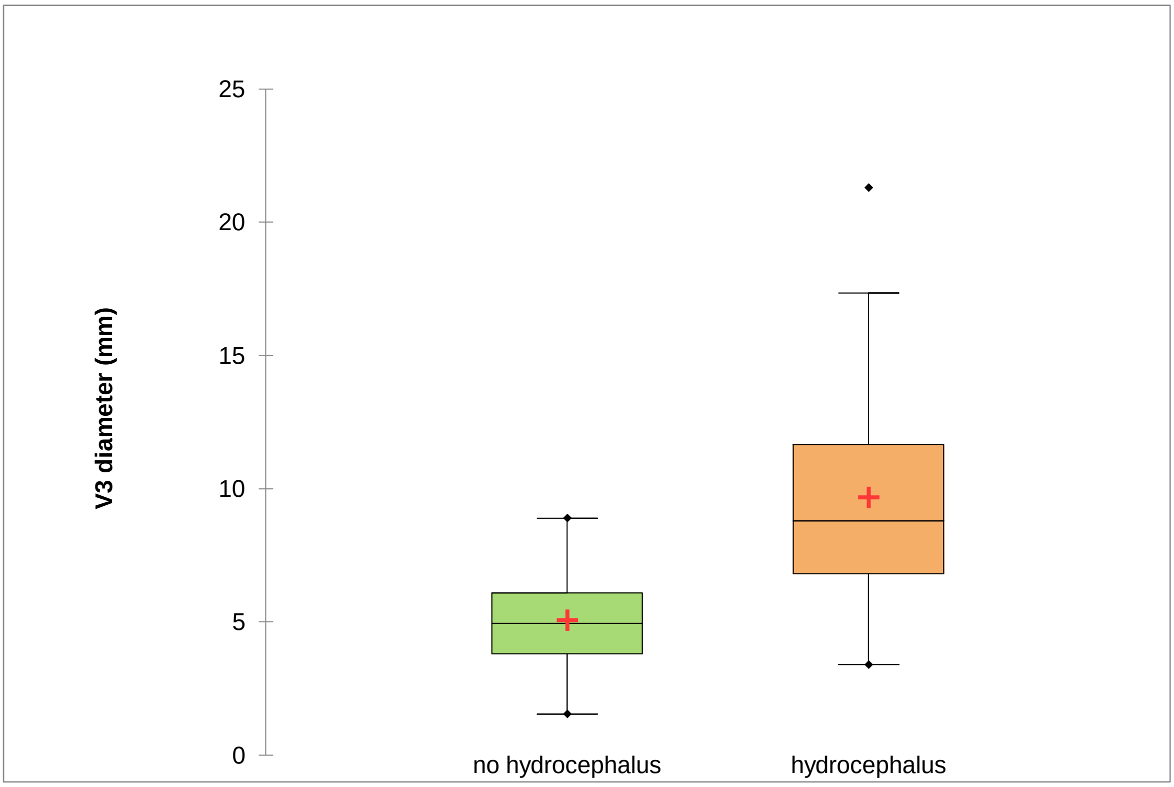

V3 diameters measured by TCS were compared in patients with or without hydrocephalus, defined by CT.

V3 diameters were analysed as the mean of the right and left TCS measurements when both sides were available, or left or right side if only one side was available.

The median V3 diameter measured by TCS was significantly greater in the hydrocephalus group (8.8 (IQR 6.8 to 11.7) mm) n=31 patients (36%), compared to the non-hydrocephalus group (5.0 (IQR 3.8 to 6.1) mm, $p<0.0001$) n=56 patients (64%).

For the box-and-whisker plots, the horizontal middle bar indicates the median, the upper and lower limits of the boxes the interquartile range, and the ends of the whiskers the 95% Confidence Interval. Spots represent minimum and maximum values. Red crosses represent the means.

Figure A5: V3 diameters measured by TCS compared with the other side

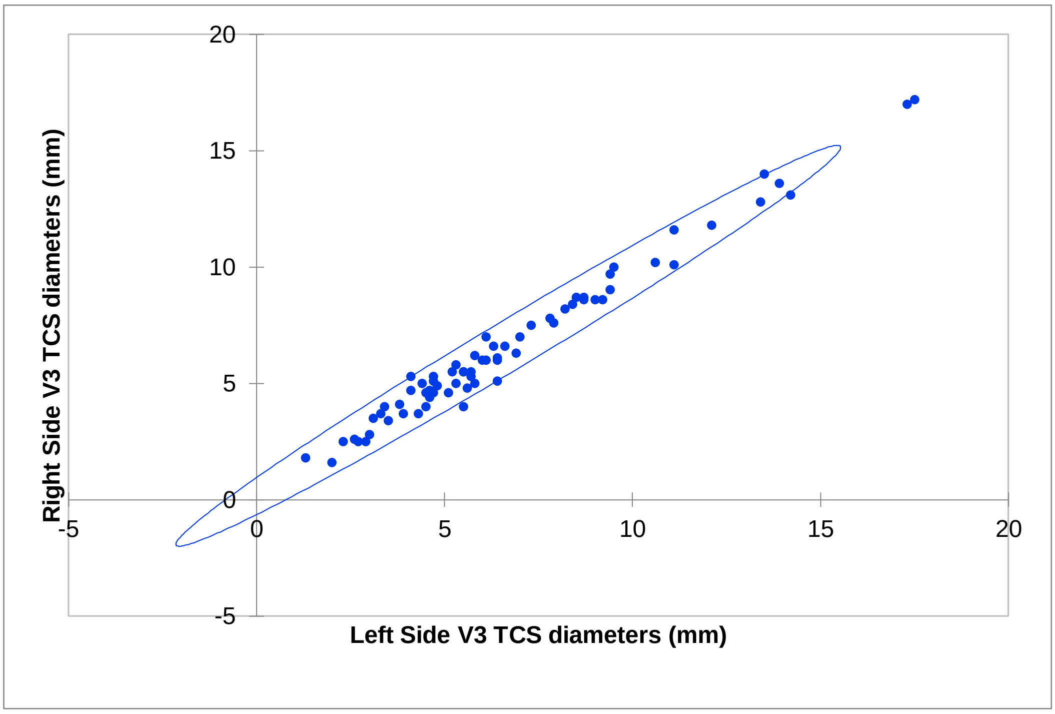

V3 diameters measured with TCS, compared with the other side. The intra-class correlation coefficient (ICC) was 0.98. Ellipse represents the Confidence Interval (CI) 95%.

CT: computed tomography; TCS: transcranial sonography; V3: third cerebral ventricle
